# Speculation on the pathophysiology of musculoskeletal injury with COVID-19 infection

**DOI:** 10.3389/fmed.2022.930789

**Published:** 2022-07-14

**Authors:** Francesca Veronesi, Deyanira Contartese, Lucia Martini, Andrea Visani, Milena Fini

**Affiliations:** Complex Structure of Surgical Sciences and Technologies, IRCCS Istituto Ortopedico Rizzoli, Bologna, Italy

**Keywords:** COVID-19, ACE2, inflammation, bone, muscle, joint

## Abstract

Coronavirus disease 2019 (COVID-19) primarily affects the respiratory tract, but also many other organs and tissues, leading to different pathological pictures, such as those of the musculoskeletal tissues. The present study should be considered as a speculation on the relationship between COVID-19 infection and some frequent musculoskeletal pathologies, in particular sarcopenia, bone loss/osteoporosis (OP) and fracture risk and osteoarthritis (OA), to hypothesize how the virus acts on these pathologies and consequently on the tissue regeneration/healing potential. The study focuses in particular on the modalities of interaction of COVID-19 with Angiotensin-Converting Enzyme 2 (ACE2) and on the “cytokine storm.” Knowing the effects of COVID-19 on musculoskeletal tissues could be important also to understand if tissue regenerative/reparative capacity is compromised, especially in elderly and frail patients. We speculate that ACE2 and serine proteases together with an intense inflammation, immobilization and malnutrition could be the responsible for muscle weakness, altered bone remodeling, increase in bone fracture risk and inflammatory joint pathologies. Future preclinical and clinical studies may focus on the regenerative/reparative properties of the musculoskeletal tissues after COVID-19 infection, toward a personalized treatment usually based on scaffolds, cells, and growth factors.

## Introduction

Coronavirus disease 2019 (COVID-19) was first detected in Wuhan, China, in December 2019 and showed a rapid spread across the world; by March 11, 2020, the World Health Organization declared a global pandemic situation. Severe acute respiratory syndrome coronavirus 2 (SARS-CoV-2) shows characteristics like the SARS-CoV, responsible for the SARS epidemic of 2002–2003 and it primarily infects the respiratory tract with pneumonia-associated symptoms, including fever, cough, difficulty breathing and fatigue (1). However, other pathological manifestations are found in other tissues of the human body including the musculoskeletal one. Therefore, in this regard, contrasting results are reported and studies are still in progress.

The aim of the present article is to investigate the relationship between COVID-19 infection and some frequent musculoskeletal pathologies, in particular sarcopenia, bone loss/osteoporosis (OP) and fracture risk and osteoarthritis (OA), to hypothesize how the virus acts on these pathologies and consequently on the tissue regeneration/healing potential, focusing in particular on the modalities of interaction of COVID-19 with Angiotensin-Converting Enzyme 2 (ACE2) and on the “cytokine storm.” Some aspects studied in this manuscript are to be considered a starting point to open a discussion and increase the orthopedic awareness on this topic.

### There are many studies on the effects of COVID-19 in the most affected tissues, but what is known about the musculoskeletal tissues?

Some clinical studies reported significantly higher mortality rate, length of hospital stay (LOS), complication rate and ventilatory need ***in COVID-19 – positive patients*** undergoing surgery because of a hip (2–6) or proximal femur fracture (7, 8). On the other side, few studies investigated the fracture risk or the bone regenerative/reparative capability in COVID-19 positive patients. Some authors showed that the incidence of frailty fractures did not differ between infected and not infected patients and the real COVID-19 effect in patients affected by fractures, especially at the hip, still remains to be ascertained (9). However, a recent study showed a suppression in cell osteogenic differentiation with a low fracture healing rate and an increase in OP in COVID-19 positive patients due to an upregulation of miR-4485 (10).

Patients infected by COVID-19 show also weakness of skeletal muscles with associated fatigue, myalgia, muscle edema, rhabdomyolysis and injury, myopathy, polyneuropathy and Guillain-Barre syndrome (11, 12). An increase in proteins of sarcoplasmatic reticulum of the muscle fibers (creatinine kinase, lactate l, alanine aminotransferase, and aspartate aminotransferase) and a decrease in pH and oxygen levels have been observed in ischemic muscle during infection (13–20). Arthralgias localized in the joints, or myalgias in muscles, have been reported in patients infected with COVID-19 (21).

Osteoarthritis, rheumatoid arthritis and reactive arthritis are also observed. This latter is an acute aseptic arthritis that occurs in few weeks after COVID-19 infection and, in the last 2 years, several articles treated this COVID-associated pathology. It involves prevalently the lower limbs with a symptoms duration up to a month and a half (22).

### What happens when the virus encounters the membrane-bound angiotensin-converting enzyme 2?

**FIGURE 1:**
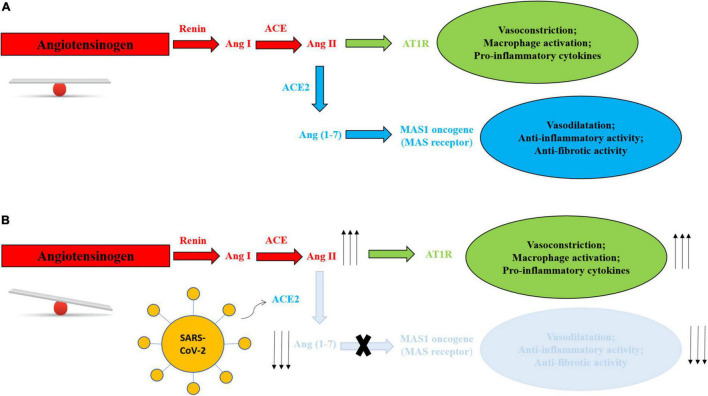
**(A)** Balanced RAS system in absence of SARS-CoV-2. **(B)** RAS system mechanism unbalance in presence of SARS-CoV-2. Virus entry leads to degradation of membranal ACE2 with depletion of Ang 1–7, blunting role of the Ang (1–7)/MAS axis and reducing its physiological and protective activities, and increasing Ang II-AT1R production and activities, with vasoconstriction, activation of macrophages and increase in inflammatory cytokines.

Angiotensin-converting enzyme 2 is used for virus entry in host cells and is a component of the ACE2/angiotensin (Ang) (1–7)/MAS axis, implicated in the regulation of body fluids, electrolytes and blood pressure. As observed in Figure 1, ACE2 converts angiotensin II (Ang II) into Ang (1–7), exerting opposite effects to Ang II (23).

Angiotensin-converting enzyme 2 expression is downregulated after the binding of the virus, blunting its role in the ACE2/Ang (1–7)/MAS axis and reducing its physiological and protective activities (24) (Figure 1).

**FIGURE 2:**
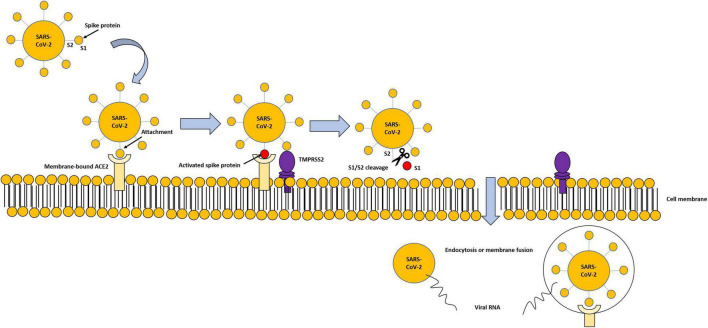
Modality of virus entry, through ACE2-TMPRSS2 interaction. Viral spike proteins enable attachment to cell-membrane-bound ACE2. TMPRSS2 attacks the S1 unit of the virus S protein and detaches S1 from the S2 unit. The viral S2 unit merges with the host cell membrane, allowing the transfer of the viral content inside the cell through endocytosis or membrane fusion, and subsequent degradation of internalized ACE2.

The entry and spread of the virus into the host cells are mediated by spike (S) protein, present on the virus surface, and facilitated by some proteases located on the host cell surface in proximity to the ACE2, such as the transmembrane serine protease 2 (TMPRSS2), a proteolytic enzyme. TMPRSS2 attacks the S1 unit of the virus S protein and detaches S1 from the S2 unit. The viral S2 unit merges with the host cell membrane, allowing the transfer of the viral content inside the cell through endocytosis or membrane fusion, ***causing all the clinical manifestation of the virus*** (Figure 2). TMPRSS2 and ACE2 are co-expressed in several tissues (25, 26).

As regard bone tissue, in healthy conditions, the relationship between osteoblasts (OBs) and osteoclasts (OCs) is perfectly balanced for the maintenance of bone mass (27). OP is one of the most important causes of morbidity and functional decline in the elderly with consequent bone fractures and bone density is reduced with an imbalance between OCs and OB activity.

Osteoblasts and OCs possess ACE2, and the axis ACE2/Ang (1–7)/MAS seems to be involved in bone remodeling and anti-inflammatory activity, increasing OB metabolism, alkaline phosphatase activity, collagen I and osteocalcin production, and keeping low the level of OC precursors. This concurs to reduce osteoclastogenesis and bone loss in healthy conditions (28, 29).

No study concerning TMPRSS2 expression in bone cells has been performed. However, one study showed that other serine proteases, observed in OBs and osteocytes, control bone mineralization, and another study demonstrated that TMPRSS2:ERG fusion is involved in prostate cancer bone metastases, resulting in bone formation increase (30). Therefore, we hypothesize that the serine protease TMPRSS2 could be present in bone and be involved in the viral infection.

Even if ACE2 concentration in skeletal muscles is lower than in other tissues, the first evidence of the presence of ACE2 in skeletal muscles appeared in 2010 (31). ACE2, through the activation of ACE2/Ang (1–7)/MAS axis, seems to be important for the maintenance of skeletal muscle physiology and activity and for the protection against muscle weakness. In animal models the blockage of ACE2 activity, through the knockout of its gene, decreases grip strength, running distance and muscle fiber size, and increases senescence-associated gene, lipid accumulation, endoplasmic reticulum stress and mitochondrial dysfunction, accelerating the muscle weakness typically observed in the elderly (31). The increase in the amount of ACE2 induces the reduction of fibrosis and inflammatory infiltration associated with muscular dystrophies (32).

Transmembrane serine protease 2 seems not to be present in the skeletal muscle tissue. Conversely, Hepsin, a type II membrane-associated serine peptidase, which TMPRSS2 is part of, is expressed at high concentration in skeletal muscle and is highly expressed in muscle tissue affected by muscular dystrophy (33).

Sarcopenia prevalently affects elderly patients and is characterized by a decline in skeletal muscle fiber mass and strength, with consequent physical activity reduction and increase in falls, disability and oxidative stress (34). Since no study has yet explored the mechanism through which COVID-19 influences sarcopenia, we can only speculate. Our hypothesis is that COVID-19 infection, with the help of serine proteases, could reduce ACE2 physiological activity exerted on the skeletal muscle, leading to muscle weakness.

There is a lack of information on TMPRSS2 and ACE2 and cartilage, however, hepsin and TMPRSS4 are expressed in this tissue, inducing collagenase expression (35). Also, other serine proteases are implicated in cartilage destruction, metalloproteinase activity, aggrecan breaking and synovial inflammation (36).

### What about inflammation, immobilization, and malnutrition?

Angiotensin-converting enzyme 2/Ang (1–7)/MAS axis cannot be assessed separately from the intense inflammation, immobilization, and malnutrition, that affect muscles and bones during COVID-19 infection.

After virus entry through ACE2, in the initial stages, cells of respiratory epithelium secrete cytokines that produce the immune response against virus, but after, when the severity of infection increases, the activated inflammatory pathways lead to the production of a storm of cytokines, the so called “cytokine storm” (37). The secretion of these pro-inflammatory cytokines leads to a dysregulation of the innate immune system, that in turn leads to the different tissue manifestations, including those of the musculoskeletal apparatus. These cytokines are considered the pathology biomarkers and are easily found in the serum of the affected patients (38).

The most important pro-inflammatory molecules produced by COVID-19 infection, that impact on musculoskeletal tissues, are interleukin-6 (IL6), IL1β, IL10, IL17, interferon-γ (INFγ), tumor necrosis factor-α (TNFα), C-reactive protein (CRP), soluble receptor for advanced glycation end products (sRAGE), receptor activator of nuclear factor-B ligand (RANKL), vascular endothelial growth factor (VEGF) and macrophage colony stimulating factor (M-CSF), that are high in serum of infected patients and induce fiber proteolysis and decrease protein synthesis. Among them, IL-6 should be considered as marker of severity of COVID-19 infection (37, 39).

In particular in bone, ACE2 has an anti-inflammatory property and reduces bone resorption. The blocking ACE2 with the virus, leads to decreased bone mass and increased joint inflammation (40).

Coronavirus disease 2019 infection increases IL6, IL1β, CXCL10, IL17, and TNFα that induce osteoclastogenesis and inhibit OB proliferation and metabolism causing increased bone fragility and risk of fractures in osteoporotic and elderly patients (41). Additionally, IL-6 is known to reduce nitric oxide (NO) bioavailability and to increase oxidative stress, leading to endothelial permeability, recruitment, and infiltration of the vascular wall by circulating leukocytes. In this context, a key aspect that cannot be underestimated is the interaction between bone tissue and vascular endothelial cells, through growth factors and chemokines. Thus, if the vascular network is damaged, the bone metabolism is also affected (42).

As regard muscles, elevated IL-6 concentration may be the sign of a vigorous immune and inflammatory response which could represent the link between severity of COVID-19 infection and muscular weakness development. IL-1β and IL-6 may cause fibrosis by inducing increased muscle fibroblast activity. IL-1β and TNF-α have been described to inhibit the differentiation and proliferation of satellite cells, the progenitor cells involved in muscle fiber growth (43).

As regard cartilage and joint, COVID-19, increasing inflammatory mediators, leads to an acceleration and worsening of OA. In addition, the clinical manifestation of reactive arthritis could be related to the increase of cytokines in the synovial fluid of patients affected by COVID-19, especially of IL17, and the activation of immune T cells. IL-1β, IL6, and TNF-α induce chondrolysis leading to arthalgia and/or the progression of OA (22).

In patients affected by COVID-19, Wingless/int1 (WNT) pathway has a role in ACE2 activity in muscles and bone. This is one of the pathways that is clearly important during skeletal development and bone homeostasis (44). In particular, after COVID-19 entry, the decrease in ACE2 activity is associated with an upregulation of WNT pathway, inducing several pathologies, such as hypertension, heart disease, and cancer (45).

There is a positive correlation between WNT/β-catenin pathway and inflammation, through an activation of nuclear factor kappa-light-chain-enhancer of activated B cells (NF-κB) pathway and an increase in Cyclooxygenase (COX), upregulated also in OA pathology (46).

Immobilization of the COVID-19 affected patients causes further proinflammatory signals that can lead to muscle and bone fragility with reduced bone mineral density (BMD) and the general hypoxia increases the production of RANKL, VEGF, and M-CSF, that activate OCs and block osteogenesis of OBs. Long duration of intensive care unit (ICU) stay, multiorgan system failure, systemic inflammatory response syndrome (SIRS), prolonged sedation, and prolonged endotracheal intubation are well-recognized risk factors for the development of neuromuscular complications (43, 47).

Finally, malnutrition as calcium deficiency is deleterious to muscle and bone tissues. Calcium is important in the muscular function, and a positive association is found between an inadequate nutritional status (low intake of protein, vitamin D, and calcium) and osteosarcopenia. Lower albumin, magnesium and calcium are also found in the serum of patients affected by COVID-19 than in healthy subjects, underling that the addition of calcium and magnesium may help to reduce the consequences of COVID-19, such as chronic fatigue-syndrome-like and physiosomatic symptoms (48–51).

## Discussion

Although other pathologies, in COVID-19 infected patients, require most of the attention of the medical community, musculoskeletal diseases and their behavior is a topic that should not be overlooked. Indeed, sarcopenia, OP and OA, alongside other comorbidities, such as hypertension, cardiac diseases, dementia, cancer and diabetes, contribute to the frailty syndrome observed in elderly patients.

Currently, it is still unclear how the effects of COVID-19 on the musculoskeletal system are mediated, and if some common pathologies (sarcopenia, OP, bone fractures, and OA) could be worsened by COVID-19 infection.

Starting from the evidence that ACE2 and TMPRSS2 are necessary for SARS-CoV-2 entry in the host cells and are co-expressed in some human tissues, we investigated on their presence in musculoskeletal cells and tissues. In addition, the extremely high inflammation cascade, which derives from and accompanies the reduction of ACE2 activity after virus entry, immobilization and malnutrition, which often accompany the patient, probably also contribute to worsening the picture of the pathologies, through the production of so many cytokines.

Given the scarcity of studies on this topic, in the future it could be important to design and carry out preclinical and clinical studies on the impact of COVID-19 infection on muscle, bone and cartilage to test in practice the theories proposed in this work. Finally, preclinical and clinical studies may focus on the regenerative/reparative properties of the musculoskeletal tissues after COVID-19 infection. An important issue could be the investigation of differences between COVID-19 infected and not infected patients at cellular, molecular and tissue levels in muscle, bone and cartilage tissues. This will be translated to personalized surgical reconstructive clinical treatments usually based on scaffolds, cells, and growth factors. Alongside the mechanisms that could contribute to the onset or worsening of a musculoskeletal pathology, the altered tissue regenerative/healing potential may also be compromised by the virus and probably could have an impact on the therapeutic surgical procedures. This could lead to an impaired post-operative progression after reconstruction and regenerative techniques.

In addition, *in vitro* studies are needed to evaluate the presence of ACE2 and TMPRSS2 in human musculoskeletal tissues because this seems to be necessary for the infection and related tissue damage.

Very few studies have been focused on the presence of ACE2 in muscle fibers, OBs, OCs and OC precursors, and no studies have been performed on ACE2 in cartilage tissue and on the presence of TMPRSS2 in the musculoskeletal tissues. In addition, few studies showed the presence of other serine proteases, of the same TMPRSS2 subfamily, in bone, muscle and cartilage; therefore, we could hypothesize that also TMPRSS2 is present in these tissues.

## Data Availability Statement

The original contributions presented in this study are included in the article/supplementary material, further inquiries can be directed to the corresponding author.

## Author contributions

FV, DC, LM, AV, and MF have made substantial contributions to the conception and design of the study, acquisition and interpretation of data, drafting of the article, and final approval of the version to be submitted. All authors contributed to the article and approved the submitted version.

## Conflict of Interest

The authors declare that the research was conducted in the absence of any commercial or financial relationships that could be construed as a potential conflict of interest.

## Publisher’s Note

All claims expressed in this article are solely those of the authors and do not necessarily represent those of their affiliated organizations, or those of the publisher, the editors and the reviewers. Any product that may be evaluated in this article, or claim that may be made by its manufacturer, is not guaranteed or endorsed by the publisher.

## Abbreviations

COVID-19: Coronavirus disease 2019
ACE2: Angiotensin-converting enzyme 2
Ang: angiotensin
TMPRSS2: Transmembrane serine protease 2
SARS-CoV-2: severe acute respiratory syndrome coronavirus 2
S: spike
OP: osteoporosis
OA: osteoarthritis
IL6: interleukin 6
OBs: osteoblasts
OCs: osteoclasts
NO: nitric oxide
LOS: length of hospital stay
IL: Interleukin
INF γ: Interferon- γ
TNF α: Tumor Necrosis Factor- α
CRP: C-reactive protein
sRAGE: Soluble Receptor for Advanced Glycation End Products
RANKL: receptor activator of nuclear factor-B ligand
VEGF: vascular endothelial growth factor
M-CSF: macrophage colony stimulating factor
NF- κ B: nuclear factor kappa-light-chain-enhancer of activated B cells
COX: Cyclooxygenase
BMD: bone mineral density
ICU: intensive care unit
SIRS: systemic inflammatory response syndrome.
